# Traveled Distance Is a Sensitive and Accurate Marker of Motor Dysfunction in a Mouse Model of Multiple Sclerosis

**DOI:** 10.1155/2013/170316

**Published:** 2013-12-10

**Authors:** Takako Takemiya, Chisen Takeuchi

**Affiliations:** ^1^Medical Research Institute, Tokyo Women's Medical University, Tokyo 162-8666, Japan; ^2^Kita Medical and Rehabilitation Center for Disabled, Tokyo 114-0033, Japan

## Abstract

Multiple sclerosis (MS) is a common central nervous system disease associated with progressive physical impairment. To study the mechanisms of the disease, we used experimental autoimmune encephalomyelitis (EAE), an animal model of MS. EAE is induced by myelin oligodendrocyte glycoprotein_35–55_ peptide, and the severity of paralysis in the disease is generally measured using the EAE score. Here, we compared EAE scores and traveled distance using the open-field test for an assessment of EAE progression. EAE scores were obtained with a 6-step observational scoring system for paralysis, and the traveled distance was obtained by automatic trajectory analysis of natural exploratory behaviors detected by a computer. The traveled distance of the EAE mice started to decrease significantly at day 7 of the EAE process, when the EAE score still did not reflect a change. Moreover, in the relationship between the traveled distance and paralysis as measured by the EAE score after day 14, there was a high coefficient of determination between the distance and the score. The results suggest that traveled distance is a sensitive marker of motor dysfunction in the early phases of EAE progression and that it reflects the degree of motor dysfunction after the onset of paralysis in EAE.

## 1. Introduction

Multiple sclerosis (MS) is the most common demyelinating disease in young adults and is associated with progressive physical impairment [1]. The most frequent clinical form is characterized by episodes of relapse and remission with multifocal demyelination in the central nervous system. To investigate the mechanisms of MS, we used experimental autoimmune encephalomyelitis (EAE), an animal model of MS. EAE is induced by myelin oligodendrocyte glycoprotein_35–55_ peptide [2–4], and the severity of paralysis in EAE is generally examined using the EAE score. EAE score is known to be useful to estimate the severity of the paralysis, but is inappropriate to detect a decline in motor activity in the early phase before appearance of definite paralysis. In addition, there is no standard scoring system for EAE signs and there is variability between research groups.

The open field test is used to evaluate the activity and anxiety of mice by measuring the walking distance of mice with normal motor function, but we are also able to quantify changes in motor dysfunction, including the progression of paralysis, by repetitive observations in a mouse model of neuromuscular disease. As mice exposed to the same open field box repeatedly over short time scales become acclimated to the environment and decrease their traveled distance slightly, the habituation effect must not be disregarded in the analysis.

In this study, we used 6-step EAE scores [4] and traveled distance in the open-field to assess the progression of EAE. We compared the time course of the traveled distance in EAE mice with the EAE score and with the traveled distance in normal mice in the early phase. In addition, we examined the relationship between the traveled distance and the EAE score after the onset of paralysis in the late phase. Then, we investigated the effectiveness in a measurement of the traveled distance as an index of EAE progression.

## 2. Materials and Methods

### 2.1. Mice

A total of 15 female 8-week-old WT mice (C57BL/6J Jcl) were used in this study; 9 WT mice were used for the induction of EAE, and 6 mice were used as naïve controls. The mice were housed four or five per cage in a room maintained at 24 ± 2°C in a standard 12 hr light-dark cycle and had ad libitum access to standard chow and water. All of the study protocols were approved by the Animal Care and Use Committee of Tokyo Women's Medical University.

### 2.2. Induction and Assessment of EAE

For EAE induction, mice were immunized with subcutaneous injections of 250 *μ*g of the myelin oligodendrocyte glycoprotein_35–55_ peptide (MOG_35–55_, MEVGWYRSPFSRVVHLYRNGK, purity > 95%, Operon Technology, Japan) in complete Freund's adjuvant (Difco, Detroit, MI, USA). In addition, the mice received intraperitoneal injections of 500 ng of pertussis toxin (Seikagaku Corporation, Tokyo, Japan) on the day of immunization and again 2 days later. The mice were observed daily, and the progression of EAE was assessed using the following scoring system: 0, no detectable signs of paralysis; 1, completely limp tail; 2, loss of the righting reflex; 3, partial hind limb paralysis; 4, complete hind limb paralysis; 5, total paralysis of the four limbs; and 6, death. The loss of the righting reflex was defined to be when the mouse had difficulty turning over after being laid on its back but exhibited no other locomotive difficulties. Mice that were scored as a 5 on two consecutive days were immediately euthanized.

### 2.3. Behavioral Recording and Analysis

Using the top scan suites (Clever Sys., Inc.), open field tests were performed on day 0 (prior to the EAE induction) and again at days 7, 10, 14, 18, and 21 after immunization for an assessment of EAE progression. Moreover, we also performed open-field tests on days 0, 7, 10, and 14 using the naïve control mice and compared the traveled distance with EAE mice to observe the habituation effect in the intervals. The mice were placed in an observation box with dimensions of 40 cm on each side with 20 cm high walls. The total activity was monitored by an overhead view video camera over a period of 2 minutes in a quiet, well-lit room. All of the behavioral tests were performed during the window from 2 p.m. to 4 p.m. under the same conditions. The natural exploratory behavior was detected by a computer, and the traveled distance was automatically calculated.

### 2.4. Statistical Analysis

The data are presented as the means ± standard error (s.e.). The statistical analyses were performed using a repeated measure ANOVA. SPSS software (SPSS Japan Inc., IBM Company) was used for analysis, and significance was determined at a *P* value < 0.05. To avoid an overestimation in the decrease of traveled distance including the habituation effect in repeated open-field tests, we need to confirm the significant difference in the traveled distance between EAE mice and naïve control mice.

## 3. Results

Here, we compared 6-step EAE scores [4] and traveled distance using the open-field test to assess the progression of EAE. The natural exploratory behaviors of the mice were detected with a video camera attached to a computer, and the traveled distance was automatically calculated. First, we studied the time course of the EAE score and the traveled distance in the early phase of EAE. Next, we compared the traveled distance in EAE mice with normal mice in the early phase. Finally, we investigated the relationship between the traveled distance and the EAE score after the onset of paralysis, which was defined by an EAE score above 0 in the late phase of EAE.

**Figure 1:**
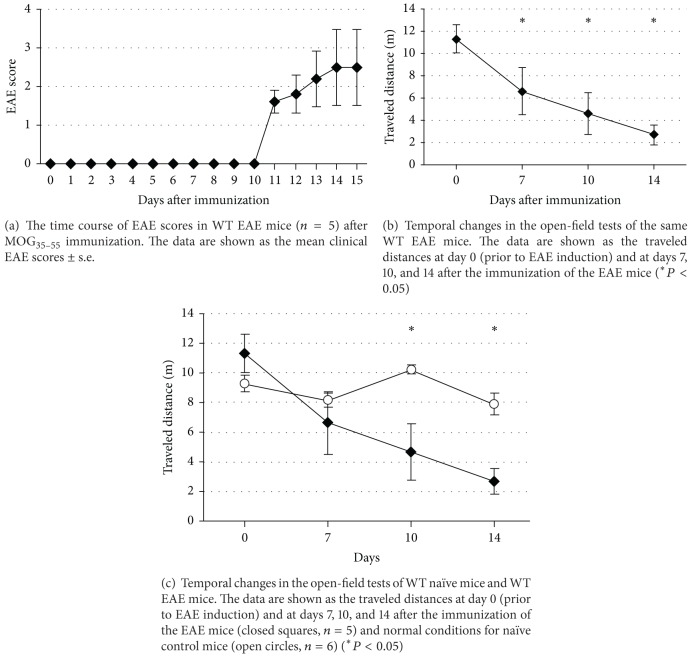
EAE progression.

The EAE scores were assessed daily to determine the development of paralysis during the first 15-day observation period following induction (Figure 1(a)). There was no increase in the EAE score until day 10, and the score gradually increased from day 11 onward. In contrast, we measured the traveled distance for 2 min in the square box on day 0 (prior to the EAE induction) and again at days 7, 10, and 14 after immunization (Figure 1(b)). We did not measure the distance every day to avoid habituation to the box. The walking distance decreased in a time-dependent fashion (Figure 1(b)), and there was a significant difference at day 7 (*P* = 0.0448), day 10 (*P* = 0.0452), and day 14 (*P* = 0.0180) compared with day 0. To confirm the absence of a habituation effect given the repetition of the open-field test, we used the same test schedule in naïve mice with the same intervals and compared the traveled distance with EAE mice (Figure 1(c)). Naïve mice showed almost no difference in their walking distance during the observation period, and there was a significant difference in traveled distance at day 10 (*P* = 0.0060) and day 14 (*P* = 0.0045) between the two groups. Besides, there is no significant difference in traveled distance at day 7 (*P* = 0.0511) between the two groups, suggesting that the decrease at day 7 includes the habituation effect.

**Figure 2:**
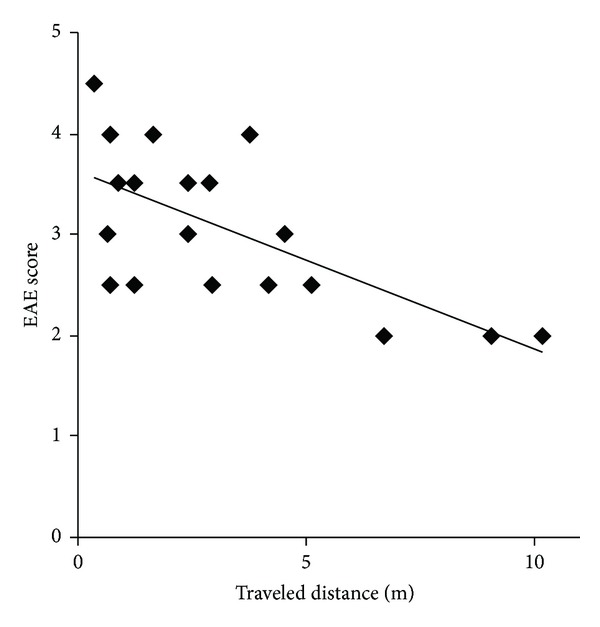
The relationship between traveled distance and EAE scores. The data are shown as traveled distances and EAE scores except in cases where EAE scores were 0 at days 14, 18, and 21 after the immunization of the EAE mice (*n* = 5–9).

Finally, we investigated the relationship between the traveled distance and definite paralysis (measured by the EAE score) at days 14, 18, and 21. Because an EAE score of 0 indicated not only no detectable signs of paralysis (slight motor dysfunction) but also normal healthy condition, the score 0 means wide variation of mice condition. Therefore, to investigate the relationship between the traveled distance and definite paralysis, the score 0 has to be excluded in this analysis. There was a high coefficient of determination between the distance and the score (*R* ^2^ = 0.438) and the relationship is shown by the following equation: *Y* = −0.177*X* + 3.625 (Figure 2). The result suggests that the measurement of walking distance is an objective tool and an accurate marker that reflects the progress of motor dysfunction after the onset of paralysis in EAE.

## 4. Discussion

The signs of EAE are caused by inflammation and demyelination of the spinal cord. The severity of EAE is generally evaluated by an EAE score (occasionally named EAE scale or grade). Mice are scored daily after the day of immunization to precisely detect the time of disease onset and to investigate the progression of the EAE. In early research in EAE, EAE scores were used with a 3- to 4-step scoring system [5, 6]. In recent years, many researchers use 0 to 5 or 0 to 6 point scales for the scoring of EAE [2, 5–18]; however, there are some problems with this method. First, each laboratory has its own method for evaluating the severity of EAE; the methods are not standardized between laboratories. Moreover, the method of assessing an EAE score depends on subjective observation. For example, researchers hold the base of the tail of mice to judge tail limpness and they touch or perform a toe pinch to evaluate the gait condition of the mice [7, 8]. In addition, how the symptom is designated “mild” or “severe” is ill-defined; thus, we need to completely remove the observer's bias in the judgment of EAE scores [5, 9–11]. Finally, the score is not a quantitative analysis and could not detect the slight motor dysfunction in the early phase before appearance of definite paralysis.

In this study, we performed quantitative behavioral analyses for the evaluation of EAE symptoms compared with the EAE score used in our laboratory [4]; therefore, we recorded videos of walking mice and automatically calculated the traveled distance. The repetitive behavioral analyses in the observation box were performed after an interval to avoid habituation effects. Then, we compared 6-step EAE scores [4] and traveled distance using the open-field test to assess the progression of EAE in the early phase and the late phase of EAE.

We found that the traveled distance was significantly decreased in the early phase of the EAE process when the EAE score showed no increase. This finding suggests that measurement of the traveled distance is a relevant tool for the assessment of motor dysfunction in the early phase of EAE progression. Because the inflammation in spinal cords causes motor dysfunction in EAE, the early decline in traveled distance might be mainly due to the motor dysfunction; nevertheless, we could not deny the participation of anxiety concerning the inflammatory process. In the future, we have to combine some behavioral analyses such as rotarod test and light-dark test to evaluate the motor dysfunction and anxiety. Furthermore, there was a high coefficient of determination between the traveled distance and the EAE score after the onset of signs suggesting that the traveled distance is an accurate and reasonable tool to evaluate motor dysfunction in the later phases of the EAE process. The MS patients often feel their legs heavy and complain about walking trouble in the early phase of human MS; thus, the measurement of walking distance within a given time might be useful to detect the motor dysfunction in MS patients.

In conclusion, our study suggests that traveled distance is a sensitive and accurate marker of early and late motor dysfunction in a mouse model of MS.

## References

[1] Noseworthy JH, Lucchinetti C, Rodriguez M, Weinshenker BG (2000). Multiple sclerosis. *The New England Journal of Medicine*.

[2] Mendel I, Kerlero de Rosbo N, Ben-Nun A (1995). A myelin oligodendrocyte glycoprotein peptide induces typical chronic experimental autoimmune encephalomyelitis in H-2b mice: fine specificity and T cell receptor V*β* expression of encephalitogenic T cells. *European Journal of Immunology*.

[3] Iglesias A, Bauer J, Litzenburger T, Schubart A, Linington C (2001). T- and B-cell responses to myelin oligodendrocyte glycoprotein in experimental autoimmune encephalomyelitis and multiple sclerosis. *GLIA*.

[4] Takeuchi C, Matsumoto Y, Kohyama K (2013). Microsomal prostaglandin E synthase-1 aggravates inflammation and demyelination. *Neurochemistry International*.

[5] Matsumoto Y, Fujiwara M (1987). The immunopathology of adoptively transferred experimental allergic encephalomyelitis (EAE) in Lewis rats. Part I. Immunohistochemical examination of developing lesions of EAE. *Journal of the Neurological Sciences*.

[6] Swanborg RH (1988). Experimental allergic encephalomyelitis. *Methods in Enzymology*.

[7] Stromnes IM, Goverman JM (2006). Active induction of experimental allergic encephalomyelitis. *Nature Protocols*.

[8] Stromnes IM, Goverman JM (2006). Passive induction of experimental allergic encephalomyelitis. *Nature Protocols*.

[9] Tsunoda I, Kuang L-Q, Tolley ND, Whitton JL, Fujinami RS (1998). Enhancement of experimental allergic encephalomyelitis (EAE) by DNA immunization with myelin proteolipid protein (PLP) plasmid DNA. *Journal of Neuropathology and Experimental Neurology*.

[10] Kalyvas A, David S (2004). Cytosolic phospholipase A2 plays a key role in the pathogenesis of multiple sclerosis-like disease. *Neuron*.

[11] Kihara Y, Matsushita T, Kita Y (2009). Targeted lipidomics reveals mPGES-1-PGE2 as a therapeutic target for multiple sclerosis. *Proceedings of the National Academy of Sciences of the United States of America*.

[12] Sobel RA, Tuohy VK, Lu Z, Laursen RA, Lees MB (1990). Acute experimental allergic encephalomyelitis in SJL/J mice induced by a synthetic peptide of myelin proteolipid protein. *Journal of Neuropathology and Experimental Neurology*.

[13] Storch MK, Stefferl A, Brehm U (1998). Autoimmunity to myelin oligodendrocyte glycoprotein in rats mimics the spectrum of multiple sclerosis pathology. *Brain Pathology*.

[14] Pollak Y, Ovadia H, Goshen I (2000). Behavioral aspects of experimental autoimmune encephalomyelitis. *Journal of Neuroimmunology*.

[15] Pollak Y, Ovadia H, Orion E, Yirmiya R (2003). The EAE-associated behavioral syndrome: II. Modulation by anti-inflammatory treatments. *Journal of Neuroimmunology*.

[16] Greter M, Heppner FL, Lemos MP (2005). Dendritic cells permit immune invasion of the CNS in an animal model of multiple sclerosis. *Nature Medicine*.

[17] Matejuk A, Hopke C, Vandenbark AA, Hurn PD, Offner H (2005). Middle-age male mice have increased severity of experimental autoimmune encephalomyelitis and are unresponsive to testosterone therapy. *Journal of Immunology*.

[18] Croxford JL, Miyake S, Huang Y-Y, Shimamura M, Yamamura T (2006). Invariant V*α*19i T cells regulate autoimmune inflammation. *Nature Immunology*.

